# Acute cholestatic hepatitis induced by Epstein–Barr virus infection in an adult: a case report

**DOI:** 10.1186/s13256-016-0859-x

**Published:** 2016-03-27

**Authors:** Anthony Khoo

**Affiliations:** Department of Medicine, The Royal Adelaide Hospital, Adelaide, South Australia 5000 Australia

**Keywords:** Cholestasis, Epstein–Barr virus, Hepatitis, Polymerase chain reaction

## Abstract

**Background:**

Acute cholestatic hepatitis without features of infectious mononucleosis is a rare presentation of primary Epstein–Barr infection, with only several cases previously reported in the medical literature. Early investigation for Epstein–Barr virus in febrile patients with deranged liver function tests and no demonstrable biliary obstruction on imaging can expedite both diagnosis and treatment, thereby avoiding costly or invasive procedures such as liver biopsy.

**Case presentation:**

A 59-year-old white woman of Anglo-Saxon descent presenting with a febrile illness was noted to have a cholestatic picture of deranged liver function tests. Over the following week a progressive obstructive jaundice developed, with no evidence of choledocholithiasis on ultrasound or magnetic resonance cholangiopancreatography. Specific immunoglobulin M antibodies against Epstein–Barr virus were detected in her serum and the diagnosis of Epstein–Barr hepatitis was confirmed by polymerase chain reaction testing. Supportive treatment was implemented and her liver function had normalized 3 months after presentation.

**Conclusions:**

Epstein–Barr virus is associated with a wide variety of clinical manifestations and can present as cholestatic hepatitis with or without features of infectious mononucleosis. While the diagnosis is often suggested by serological testing, Epstein–Barr virus polymerase chain reaction is a new non-invasive laboratory study that can help identify infection in cases where the clinical presentation is atypical.

## Background

Epstein–Barr virus (EBV) is a ubiquitous human herpesvirus with a worldwide prevalence in the adult population that approaches 90 %, the majority of infections being both subclinical and inapparent. Transient increases in hepatic transaminases during primary infection are not uncommon but significant icterus and cholestasis are rare, with an incidence of less than 5 % [1]. Only several cases of EBV-induced cholestasis without features of infectious mononucleosis such as pharyngitis and glandular swelling have been previously reported in the medical literature [2].

Primary EBV infection can be identified by the presence of immunoglobulin M (IgM) antibodies directed against the Epstein–Barr viral capsid antigen (VCA). Anti-VCA immunoglobulin G (IgG) is produced concurrently early in infection (day 4 to 7) and persists for the lifetime of an immunocompetent person [3, 4].

EBV polymerase chain reaction (PCR) is a new non-invasive test which can assist in diagnosing primary infection, particularly when the presentation is atypical. Furthermore, PCR is more sensitive than antibody testing in early stages of EBV infection [5]. It should be noted, however, that although it has diagnostic value, the quantitative aspect of EBV-PCR in immunocompetent individuals remains uncertain and the role of monitoring viral load to measure progression is yet to be validated [4].

## Case presentation

A 59-year-old, previously well, white woman of Anglo-Saxon descent was admitted to our hospital with a 4-day history of intermittent fevers, rigors and chills. She denied pain, shortness of breath, cough or urinary symptoms. There was no history of illicit drug use, recent travel or infective contacts. Her past medical history included a previous cholecystectomy and anxiety for which she was medicated with 200 mg desvenlafaxine.

On presentation, she was febrile at 39.2 °C, had a pulse rate of 80 beats/minute, blood pressure of 130/64 mmHg, respiratory rate of 14 breaths/minute and oxygen saturations of 99 % on room air. There was no visible rash, lymphadenopathy or evidence of pharyngitis. Her cardiovascular, respiratory and abdominal examinations were unremarkable.

Although her initial full blood count indices were within normal limits, biochemistry revealed a mixed picture of deranged liver function tests (LFTs) as shown in Table 1.

**Table 1:** Initial liver function tests

Test	Results	Units	Reference range
Bilirubin	37	umol/L	6–24
GGT	466	U/L	0–60
ALP	342	U/L	30–110
ALT	728	U/L	0–55
AST	460	U/L	0–45

*ALP* alkaline phosphatase, *ALT* alanine transaminase, *AST* aspartate aminotransferase, *GGT* gamma-glutamyl transpeptidase

A repeat examination the following day revealed marked icterus in addition to generalized abdominal discomfort and myalgia, with biochemistry showing progressively worsening cholestatic LFTs and conjugated hyperbilirubinemia, peaking on the seventh day of admission (see Table 2).

**Table 2:** Liver function tests after one week

Test	Results	Units	Reference range
Bilirubin	155	umol/L	6–24
GGT	536	U/L	0–60
ALP	775	U/L	30–110
ALT	364	U/L	0–55
AST	275	U/L	0–45

*ALP* alkaline phosphatase, *ALT* alanine transaminase, *AST* aspartate aminotransferase, *GGT* gamma-glutamyl transpeptidase

Her albumin was 25 g/L (34–48) and coagulation studies were within normal limits. Full blood count and blood film demonstrated a reactive lymphocytosis: 7.34×10^9^/L; reference range (RR) 1.5–3.5×10^9^/L.

Viral studies revealed a positive EBV IgM and IgG antibody. No hepatitis A IgM antibodies were detected. Hepatitis B and C serology was negative. Smooth muscle and mitochondrial antibodies were not detected. An abdominal ultrasound revealed no evidence of intra- or extrahepatic duct dilatation or choledocholithiasis. Magnetic resonance cholangiopancreatography (MRCP) demonstrated an enlarged spleen at 16 cm but was otherwise unremarkable. EBV-PCR was performed detecting 68,300 copies of EBV-ribonucleic acid (RNA) and a diagnosis of acute cholestatic hepatitis secondary to Epstein–Barr infection was subsequently made.

She was managed supportively, with paracetamol 1 g four times daily to help control pyrexia and a short course of cholestyramine 4 g three times daily to assist in managing intractable pruritus. Her clinical condition gradually improved and on review 3 months after initial admission her LFTs had returned to within normal limits.

## Discussion

Investigation of an obstructive pattern of LFTs rests heavily on imaging, as intra- and extrahepatic cholestasis cannot reliably be differentiated on the basis of biochemical results alone. Ultrasonography is a cost-effective, minimally invasive technique which can demonstrate gallstone disease and suggest extrahepatic cholestasis by revealing biliary dilation, while MRCP is a more powerful tool reserved for cases in which ultrasound imaging is inconclusive.

Once biliary obstruction has been excluded, intrahepatic causes of cholestasis should be investigated. Common hepatocellular diseases characterized by hyperbilirubinemia and a cholestatic picture of LFTs include viral infections and drugs such as phenytoin or amiodarone. Primary biliary cirrhosis and cholestasis of pregnancy are less common causes which can be readily excluded while infiltrative disorders such as sarcoid, lymphoma and amyloidosis are rare.

In addition to EBV, other viral causes of intrahepatic cholestasis include cytomegalovirus and viral hepatitis A, B, C and E. Acquired immune deficiency syndrome (AIDS) cholangiopathy is a syndrome of biliary obstruction resulting from infection-associated stricturing of the biliary tree, usually seen in patients with a CD4 count below 100/mm^3^.

While the pathogenesis of cholestasis in EBV infection has not been well defined, it is thought to be primarily an immune-mediated rather than cytotoxic phenomenon [5]. Proposed mechanisms of cholestatic hepatitis in EBV include inflammation and swelling of the bile duct or direct damage to hepatic cells by autoantibody-mediated activation of free radicals [5–7].

The management of EBV cholestasis is focused on supportive measures and there is limited data supporting the use of corticosteroids or antivirals in severe EBV infections [8]. Symptomatic measures can include the use of simple analgesics and paracetamol to lower fever or a trial of cholestyramine in cases of intractable pruritus.

Keeping EBV hepatitis in the differential for febrile patients with a cholestatic picture of LFT derangement can not only expedite accurate diagnosis and treatment but may also potentially avoid further costly or invasive diagnostic procedures such as liver biopsy and endoscopic retrograde cholangiopancreatography.

## Conclusions

Epstein–Barr-induced cholestatic hepatitis is an important diagnosis to consider in the patient presenting with fever and deranged LFTs, particularly when no demonstrable cholelithiasis is present on imaging. While diagnosis can usually be obtained through serological testing, EBV-PCR is a new technique with high sensitivity and specificity that can confirm Epstein–Barr infection in cases where clinical presentation is atypical or serology inconclusive.

## Consent

Written informed consent was obtained from the patient for publication of this case report and accompanying images. A copy of the written consent is available for review by the Editor-in-Chief of this journal.

## Abbreviations

AIDS: Acquired immune deficiency syndrome
ALP: Alkaline phosphatase
ALT: Alanine transaminase
AST: Aspartate aminotransferase
EBV: Epstein–Barr virus
GGT: Gamma-glutamyl transpeptidase
IgG: Immunoglobulin G
IgM: Immunoglobulin M
LFT: Liver function test
MRCP: Magnetic resonance cholangiopancreatography
PCR: Polymerase chain reaction
RNA: Ribonucleic acid
RR: Reference range
VCA: Viral capsid antigen
